# The Continued Significance of Obstetric Violence: A Response to Chervenak, McLeod-Sordjan, Pollet et al

**DOI:** 10.1089/heq.2024.0093

**Published:** 2024-08-07

**Authors:** Dána-Ain Davis, Monica J. Casper, Evelynn Hammonds, Wendy Post

**Affiliations:** ^1^ Department of Urban Studies, Queens College, CUNY, New York, New York, USA.; ^2^ Department of Sociology, San Diego State University, San Diego,California, USA.; ^3^ Department of the History of Science, Harvard University, Cambridge, Massachusetts, USA.; ^4^ School of Nursing, The George Washington University, Ashburn, Virginia, USA.

**Keywords:** obstetrics, obstetric violence, medical racism, Black women, reproductive health, childbirth

## Abstract

This guest editorial offers a critical response to Chervenak, McLeod-Sordjan, Pollet et al.’s clinical opinion dismissing obstetric violence as both emotionally charged and damaging to provider–patient relationships. We assert that obstetric violence remains a significant and useful framework to name and challenge racist, misogynist, and harmful medical practices. We note that such harmful practices are embedded in systems and cannot be addressed merely by individual physicians or shifts in the provider–patient relationship. Throughout, we situate the term obstetric violence in historical and legal context and demonstrate its continuing relevance to contemporary reproductive health care.

In a recent clinical opinion article in the *American Journal of Obstetrics and Gynecology*, Chervenak, McLeod-Sordjan, Pollet et al. argue that the use of the term obstetric violence is “not only a clinical false descriptor but also a political rhetoric.”^1^ Describing obstetric violence as an “emotionally charged” term, they suggest that it “increases the degree of conflict between the patient and the provider who may disagree about the best course of treatment and may also vilify the provider as an intentional perpetrator of interpersonal violence.”^1^ They prefer the use of the term “mistreatment” as both more capacious and more accurate, suggesting that “it encompasses all aspects of abuse and mistreatment.”^1^

The term violence has several synonyms, including inhumanity, heartlessness, and cruelty.^2^ And while the authors deploy the World Health Organization (WHO) definition of violence that is capacitated by intention, the WHO defines violence against women as “any act of gender-based violence that results in, or is likely to result in, physical, sexual, or mental harm or suffering, including threats of such acts, coercion or arbitrary deprivation of liberty whether occurring in public or in private life.”^3^ This is an important point because in the gendered dimensions of violence (here we are using the term gendered broadly to capture women and those people who give birth), intention is not a defining feature of violence. We therefore believe that the term obstetric violence is appropriate to describe the experiences of women and birthing people, particularly given that they themselves describe their encounters as such.^4,5^

Moreover, though the authors frame their argument as a “study,”^1^ it is unclear what their research methodology is beyond a literature review—and a careless one at that. Several key concepts they mention, such as obstetric racism,^6^ are inadequately referenced, and their reading of the obstetric violence literature is both selective and biased. Chervenak, McLeod-Sordjan, Pollet et al. are political rhetoric masquerading as objective truth.

The term *obstetric violence* in its current usage was coined in Venezuela in 2007, as part of the “Organic Law on the Right of Women to a Life Free of Violence.”^7^ In the context of widespread violence against women globally and the urgent need for protection and remediation, obstetric violence is defined as “the appropriation of the body and reproductive processes of women by health personnel, which is expressed as dehumanized treatment, an abuse of medication, and to convert the natural processes into pathological ones, bringing with it loss of autonomy and the ability to decide freely about their bodies and sexuality, negatively impacting the quality of life of women.”^7^ In contrast, Chervenak, McLeod-Sordjan, Pollet et al. trace the first use of the term to an 1827 *Lancet* article, which described “forceful removal of the placenta.”^1^ Whereas they note that the term’s current framing was designed to help foster recognition of violence against women, they “strongly disagree.”^1^ They remind readers that obstetricians “champion” and “empower” women, and that it is “inappropriate to define ‘obstetric violence’ as a form of structural violence that permeates sociopolitical contexts.”^1^

Legal scholar Elizabeth Kakura and others have focused attention on the gendered aspects of obstetric violence.^8,9^ Women and transgender or nonbinary people with uteruses are the categories of people who usually become pregnant, either willingly or not. Obstetric violence combines the gender-specificity of pregnancy, deep-seated distrust of female bodies, paternalistic medicine, and fetal harm mitigation in what sociologist Monica J. Casper calls “a combustible brew that targets women both unfairly and dangerously.”^10^ As human rights attorney Farah Diaz-Tello states, “the gender bias underpinning the use of threats and coercion to enforce medical advice is not subtle. It is axiomatic that a person of sound mind cannot be forced to undergo a medical procedure … even if the procedure would save the life of another person and even if that other person were their child. Pregnant women, however, are expected to sacrifice their health and dignity, and even potentially their lives, in the name of having a healthy baby.”^11^

Obstetric violence is recognized legally in some jurisdictions as a specific form of violation of women’s rights during childbirth, characterized by abusive medical practices, dehumanizing treatment, and coercion by health care providers.^8^ The term encompasses a variety of misconducts, including unnecessary cesarean sections, episiotomies without consent, refusal to administer pain medications, and a host of other actions that disregard the autonomy, dignity, and rights of women.^11–15^ Using a legal definition of obstetric violence that recognizes “maternity care in the United States is in a state of crisis”^8^ can help to ensure that such behaviors are not only deemed unethical but also illegal. A legal framework can support the development of clearer guidelines for practitioners and stronger protections for pregnant and birthing people, potentially leading to changes in hospital policies, regulatory statutes, and public awareness campaigns focused on upholding the dignity of all women during childbirth.

Anthropologist Dána-Ain Davis coined the term *obstetric racism* to describe “the contours of racism that materialize during Black women’s medical encounters.”^6^ She writes, “Obstetric racism is not new, but rather, it is entangled with histories that shadow contemporary expressions of medical racism deployed on Black women’s bodies. The way that Black women have been demonized, stereotyped, violated, and policed in the past is consistent with contemporary medical interactions and operate as reminders of that past.” In disregarding the term obstetric violence, Chervenak, McLeod-Sordjan, Pollet et al. simultaneously disregard obstetric racism and devalue the experiences of women of color, especially Black women, in the U.S. health care system. The importance of understanding obstetric racism is to hold the medical system, writ large, accountable for its historical role in contributing to the uneven reproductive outcomes we have today.^16^ Numerous scholars have documented abuses ranging from overutilization of cesarean section (c-section) to diagnostic lapses such as not identifying hemolysis with a microangiopathic blood smear, elevated liver enzymes, and low platelets in pregnant and postpartum patients (HELLP) syndrome.^17^ These abuses factor into the nation’s comparatively high maternal morbidity and mortality rates, with Black women disproportionately represented.^18^ While not ignoring racism completely—the authors mention it in one paragraph as “mistreatment”—Chervenak, McLeod-Sordjan, Pollet et al. nonetheless engage in what scholars Frank Harris III and J. Luke Wood term *racelighting*.^19^

They also perpetuate what anthropologist Chelsey Carter calls “anti-Black medical gaslighting”^20^—disregarding women patients’ experiences in much the same way that survivors of gender-based violence are routinely disbelieved and revictimized. Indeed, Chervenak, McLeod-Sordjan, Pollet et al. offer a master class in how professionals rely on their own “expert” knowledge to disregard and reframe the lived experiences of people with less power than themselves. They position obstetrics as ethical in its intentions and therefore harmless in its outcomes, conflating intention with outcome. For example, they describe obstetrics as “dedicated,” “well-founded,” and “humanized” while simultaneously describing various forms of abuse as “mistreatment.”^1^ We contend that Chervenak, McLeod-Sordjan, Pollet et al. cannot have it both ways. It seems their interest in disregarding obstetric violence as a useful term is primarily focused on maintaining an ideal typology of the provider–patient relationship and not on patients’ experiences of harm. It is precisely these harmful experiences, recounted to the media, scholars, and advocates, that led to the innovation of the term obstetric violence in the first place.^21,22^

In an especially viscerally disturbing example of obstetric violence, a lawsuit alleges that during a complicated delivery at Georgia’s Southern Regional Medical Center, an infant was tragically decapitated.^23^ Compounding this horror, the medical staff reportedly tightly swaddled the infant in blankets to conceal the decapitation and then displayed the baby to the family through the nursery window, misleading them about the fatal injuries. The truth emerged only after the mortuary intervened and contacted law enforcement.^23^ This grave incident underscores the critical need for systemic reforms to prevent such egregious acts and to ensure that maternal health settings maintain stringent standards of accountability and transparency.

In naming the various forms of obstetric harm—including physical and verbal abuse, nonconsensual procedures, discrimination and stigmatization, neglect, and separation of babies from mothers—as “mistreatment” rather than as violence, Chervenak, McLeod-Sordjan, Pollet et al. attempt to sanitize and neutralize dangerous and discriminatory practices. Such language deflects responsibility from individuals and institutions that perpetuate harm and ignores history.^24^ If, as the authors suggest, the term “violence” exaggerates harm (which we do not believe), the term “mistreatment” grossly minimizes harm. We ask, what are the clinical, ethical, and political stakes of minimizing patient harm? Why is it important for Chervenak, McLeod-Sordjan, Pollet et al. to diminish the harm to patients, especially women of color, who have experienced obstetric violence? Who benefits when violence is not recognized and named *as* violence?

It is worth noting here that several of the authors are connected to Lenox Hill Hospital in New York. Black women in New York City are nine times more likely to die as a result of childbirth.^25^ According to a New York Department of Health maternal mortality and morbidity report published in 2022, there were 57 pregnancy-associated deaths in New York City in 2019, out of 106,097 births. Twenty-six of these women were Black, and 20 others were Latina.^26^ Nationally, the maternal morbidity and mortality rates for women of color, especially Black women, are so concerning that, in 2020, Black women in the United States were three times more likely to die from pregnancy-related causes than White women, according to the Centers for Disease Control and Prevention. This stark disparity has prompted legislators to advance comprehensive measures such as the Black Maternal Health Momnibus Act of 2021,^27^ aimed at tackling the social determinants impacting maternal health outcomes and reducing the racial disparities in maternal health care.

For example, Beyoncé’s childbirth experience at Lenox Hill Hospital, as detailed in her candid interview with *Vogue*, sheds light on harmful practices within the health care system, even at elite hospitals, that transcend economic barriers.^28^ In the interview, she opened up about her intense and difficult childbirth experience, which involved “toxemia and an emergency c-section after her health and that of her twins was at risk.” Despite her status and access to what many would assume is the best medical care available, her narrative included emotional and physical trauma, a reality faced by many Black women, regardless of their socioeconomic status. “I was in survival mode and did not grasp it all until months later,” she remarked, noting that the seriousness of undergoing a c-section is still not fully understood.^28^ Beyoncé’s account of her pregnancy complications and the pressures of postpartum recovery reveals that even affluent Black patients are not immune to systemic shortcomings and potential biases in maternity care.^29^

Obstetric violence at Lenox Hill Hospital extends beyond direct physical mistreatment into realms of systemic neglect and policy failures, particularly evident in their approach to Medicaid and mental health care. Lenox Hill Mind Care, while providing critical psychiatric services like medication management and therapy, notably does not accept Medicaid. This exclusion is significant as Medicaid is crucial for many low-income women, offering essential coverage that includes psychiatric care. Considering that up to 20% of mothers suffer from postpartum depression,^30^ timely access to mental health services is essential for preventing long-term adverse effects on both mother and child. The denial of Medicaid at Lenox Hill indirectly perpetuates systemic violence by withholding necessary mental health support during a pivotal period in a woman’s life, exacerbating the challenges for the most vulnerable populations.

Furthermore, disparities in clinical practices at Lenox Hill Hospital, as highlighted by the Leapfrog Hospital Survey, add another layer of concern. The hospital episiotomy rate stands at 7.8%, notably above the national target goal of 5%, and their cesarean section rate is 27%, which is higher than rates observed at hospitals like North Central Bronx Hospital—a facility in a borough known for troubling birth outcomes. In the United States in 2022, the rate of primary cesarean section deliveries was 22.5 per 100 live births.^31^ These statistics not only reflect on Lenox Hill’s clinical practices, but they also highlight a broader issue of economic and procedural barriers that hinder access to essential, quality health care services. By comparing these outcomes with other local hospitals, such as Harlem Hospital and North Central Bronx Hospital, it is clear that Lenox Hill exhibits discrepancies that could be indicative of deeper, systemic issues within the hospital’s operational and policy frameworks. This reality, combined with the institution’s Medicaid policy, paints a concerning picture of how economic barriers and hospital policies can impact the quality of care and outcomes in maternal health services.

We do not disagree wholly with Chervenak, McLeod-Sordjan, Pollet et al. For example, we appreciate their obvious desire to improve obstetric care and to recognize that abuse and maltreatment exist. We also believe they may care deeply for their patients. Yet individual physicians caring for their patients cannot overcome systemic problems and injustices, including the absence of universal health care that disproportionately impacts minoritized and low-income communities. We wish the authors had approached their aims without positioning obstetric violence as the so-called “straw woman” against which they advanced their argument. The mere use of the term obstetric violence is not, as they assert, a danger to the provider–patient relationship. Structural inequities, including misogyny and racism, are the real dangers to provider–patient relationships. In disavowing obstetric violence, and by extension obstetric racism, as a lived experience for so many, hospitals obscure and refuse to capture data that express the actual encounters people have. But in understanding the scope of the problem, which can be assessed using validated tools such as the PREM-OB Scale of Obstetric Racism,^32^ created by OB/GYN and epidemiologist Karen A. Scott, quality of care can be both implemented and improved.

Sadly, Chervenak, McLeod-Sordjan, Pollet et al., like so many physicians, seem unaware of the long history of medical racism in this country. “There has never been a time in this country without racial health disparities.”^33^ The historical record shows that the treatment of Black women in medicine, specifically in obstetrics and gynecology, is largely one of horrific abuse and neglect that has been deeply embedded in medical theory and practice for nearly 400 years.^34^ This is not a history of individual physicians lacking in attempts to provide compassionate care or simple mistreatment of their patients. It is, rather, a history that shows the persistence of systemic racism in medicine and how it accounts for the chronically high maternal morbidity and mortality rates of Black women. Yet, these disparities have become so normalized that it is difficult for some analysts and practitioners to see how racism shapes “… power relations between institutions and actors and their health consequences.^”35^ Historians of medicine have charted how Black people were “… isolated and removed from the world of health and locked within a prison of sickness over the course of the 20th century.”^36^ A lack of awareness of this complex history and its legacies can itself lead to mis-naming and under recognition of racism and its impacts, furthering obstetric violence and harm.

## Abbreviations Used

HELLP: Hemolysis, Elevated Liver enzyme levels, and Low Platelet levels
OB/GYN: obstetric/gynecologist
WHO: World Health Organization
